# The Role of Human Papillomavirus in Human Immunodeficiency Virus Acquisition in Men who have Sex with Men: A Review of the Literature

**DOI:** 10.3390/v4123851

**Published:** 2012-12-18

**Authors:** Brandon Brown, Mariam Davtyan, Jerome Galea, Erica Chow, Segundo Leon, Jeffrey D. Klausner

**Affiliations:** 1 University of California, 653 E. Peltason Road Suite 2024, Irvine 92697, CA, USA; E-Mails: mdavtyan@uci.edu (M.D.); eechow@uci.edu (E.C.); 2 Epicentro, Jr. Jaén 250 A, 4, Lima, Peru; E-Mail: jgalea@epicentro.org.pe; 3 Universidad Nacional Mayor de San Marcos Instituto de Medicina Tropical, Av. Germán Amézaga s/n. Ciudad Universitaria, 1, Lima, Peru; E-Mail: srleons@yahoo.com; 4 UCLA Program in Global Health, 9911 West Pico Blvd., Suite 955, Los Angeles 90035, CA, USA; E-Mail: jdklausner@mednet.ucla.edu

**Keywords:** human papillomavirus, human immunodeficiency virus, men who have sex with men, HIV incidence, review, HPV testing

## Abstract

Human Papillomavirus (HPV) infection is the most common sexually transmitted infection (STI) worldwide. Incidence rates of HPV infection among human immunodeficiency virus (HIV)-infected individuals are well documented and are several-fold higher than among HIV-uninfected individuals. Few studies have demonstrated an increased risk for acquiring HIV infection in those with HPV infection, and this risk seems to be higher when HPV strains are of high-risk oncogenic potential. The estimated prevalence of high-risk oncogenic HPV infection is highest in men who have sex with men (MSM), a particularly vulnerable group with high prevalence rates of HIV infection and other STIs. In this paper, we provide a comprehensive review of the available literature on the role of HPV infection in HIV acquisition. Our review includes data from cross-sectional and longitudinal studies.

## 1. Introduction

HPV is the most common STI [1],that affects majority of sexually active persons at some point in their lifetime. While HPV is largely a transient infection with high rates of spontaneous clearance, persistent infection with low risk oncogenic and high risk oncogenic HPV genotypes can lead to extragenital warts and precancerous anogenital conditions [1], such as low and high grade intraepithelial lesions [2], and potentially life-threatening conditions such as cancers of the cervix [3], anus [3,4,5,6] and throat [7]. Between 22 and 34% of men who have sex with men (MSM) carry high-risk HPV genotypes which not only might increase rates of anogenital malignancies, but also HIV acquisition [1,2,3,8]. Furthermore, among MSM, there is a 37-fold increase in relative risk [8] and 44 times higher probability [3] of anal cancer compared to the general population. Current literature indicates that more than 80% of MSM who acquire HIV-infection have anal HPV infection [9]. While HPV is highly prevalent in both HIV-uninfected and HIV-infected MSM, some studies suggest that acquisition of HIV by HIV-uninfected MSM may be facilitated by the presence of high-risk HPV genotypes [1,3,10]. High-risk HPV includes genotypes 16, 18, 31, 33, 34, 35, 39, 45, 51, 52, 56, 58, 59, 66, 68, and 70 [7]. Goldstone *et al* [11], in their study of 602 MSM, reported a combined prevalence rate of 48.1% of DNA detection for any tested HPV type (HPV 6, 11, 16, 18, 31, 33, 35, 39, 45, 51, 52, 56, 58, and 59) at enrollment. Fifty-seven subjects (9.6%) were DNA positive to 4 or more of the 14 tested HPV types at enrollment in any of the external genital and/or anal swabs, and the majority of these cases were related to multiple infections in the anal canal (46 [7.8%]). HPV types 16, 6, and 56 were the most common anal infections detected, found in 13.7% (n = 81), 13.4% (n = 79), and 12.4% (n = 73) of subjects, respectively. Van Der Snoek *et al* [12] found that in HIV-negative men, HPV-6 was the most frequently detected HPV subtype, and was detected 30 times (12.4%) in anal specimens and 12 times (5.0%) in coronal sulcus specimens.

Studies estimate that around 40% of MSM carry more than one high-risk oncogenic HPV genotypes [13] which further increases their likelihood of acquiring HIV infection [1]. The proposed mechanisms are that HPV-related high-grade anal lesions resulting from HPV genotypes with oncogenic potential increase tissue microvasculature, friability, recruitment of CD4+ T cells and dendritic cells, and activate the cell mediated immune response leading to higher susceptibility to HIV infection [1]. Additionally, it has been demonstrated that the E7 protein of HPV type-16 down-regulates the expression of a molecule called E-Cadherin, whose function is adhesion, potentially increasing susceptibility of the genital lining to HIV infection. The genital lining consists of specialized cells called Langerhans’ cells (LC), which can internalize HIV as a result of HPV-related morphological changes and reduced density [10]. Other important risk factors for HPV infection in MSM include co-infection with other STIs such as syphilis and Herpes Simplex Virus type 2 (HSV-2) which produce lesions that may destroy the integrity of anogenital epithelial cells and facilitate HIV invasion [14], engagement in strictly receptive anal intercourse *vs.* insertive [11], seeking sex partners in gay venues [10], methamphetamine use, having a sexual partner who injects drugs [5], smoking, exchanging sex for money [15], and HIV infection [16].

Studies also indicate that MSM infected with high risk HPV genotypes are more likely to be infected with other STIs such as rectal infection with either *Chlamydia trachomatis or Neisseria gonorrhoeae* [15], as well as HSV-2 [10], thereby increasing their vulnerability to HIV infection. While a substantial number of research protocols have examined the association between STIs and HIV infection among MSM, few have explored the role of HPV on HIV and the incidence of other STIs [6]. For instance, MSM infected with high risk anal HPV are more likely to be co-infected with HSV -2, *Gonorrhea*, *Chlamydia* , and HIV [11]. Similarly, MSM with high risk HPV are more likely to have participated in unprotected receptive anal intercourse leading to increased transmission and hence increased incidence rates of HPV, HIV and other STIs [11]. Pando *et al* [6] reported higher prevalence rates of HIV, Hepatitis B Virus (HBV), *Treponema Pallidum* (Syphilis), and HPV among MSM and these results were statistically significant. Quinn *et al* [15] reported that rectal bacterial infection was significantly associated with high-risk HPV type infection suggesting that high-risk behaviors increase the risk for rectal infections and susceptibility to HPV with oncogenic potential. Zhang *et al* [14], in their study of Chinese MSM reported 19.2% prevalence rate of syphilis and 71.4% prevalence rate of anal HPV indicating that unprotected sexual behaviors are common in MSM and substantially increase the potential for HIV transmission. The authors also observed significantly higher rates of HIV among those co-infected with syphilis and anal HPV. 

## 2. Results and Discussion

### 2.1 Results

We conducted a review of the literature by searching PubMed using the terms “human papillomavirus” or “HPV”, “HIV” or “human immunodeficiency virus”, and “MSM” or “men who have sex with men” to find articles published from September, 1998 to August, 2012. Studies were included irrespective of testing methods used for HIV and HPV diagnosis. We excluded studies without HPV data from both HIV-infected and HIV-uninfected individuals. We also excluded studies that were not written in English, or opinion papers, commentaries, case reports, and subsamples from original papers. Additionally, we identified significant references in review papers and subsequently excluded the review paper itself.

**Figure 1:**
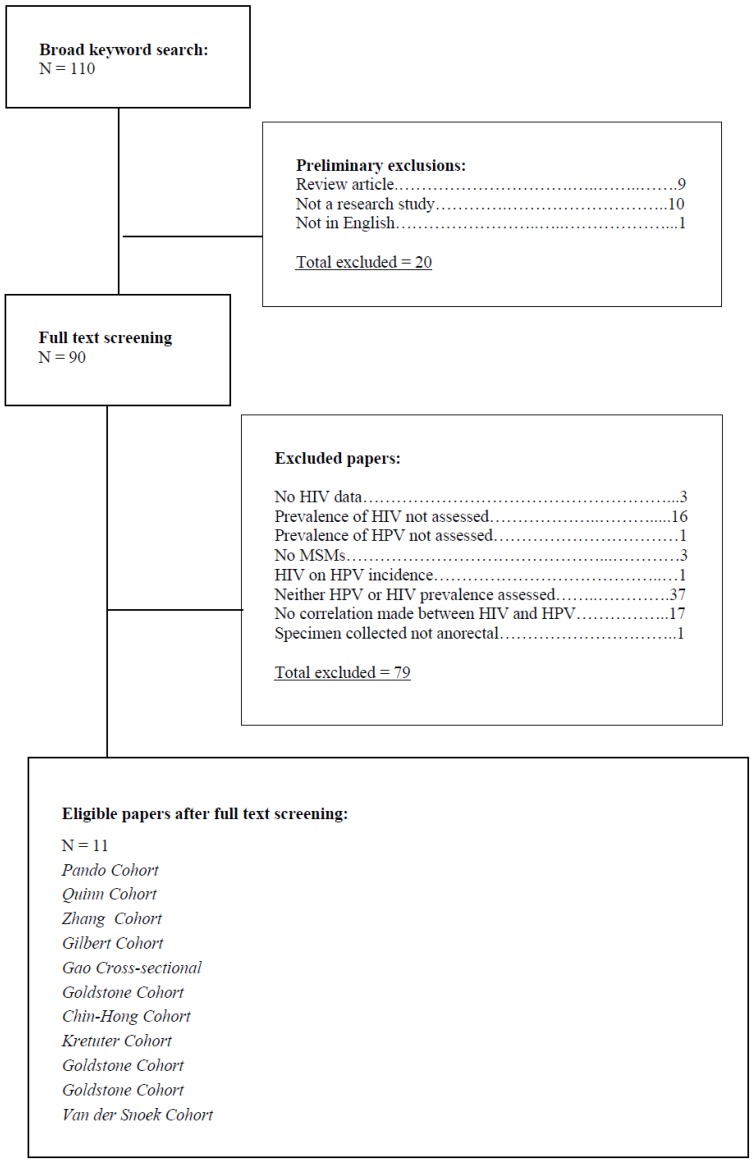
Human Immunodeficiency Virus (HIV) Human Papillomavirus (HPV) flowchart of articles.

Our search criteria identified 110 articles of which 20 were eliminated because they were reviews of published research (n = 9), were not research studies (n = 10), and not written in English (n = 1). Of the 90 remaining articles we eliminated 79 because they had no data on HIV (n=3), HIV prevalence was not assessed (n = 16), HPV prevalence was not assessed (n = 1), MSM were not the target population (n = 3), the effect of HIV on HPV incidence was assessed (opposite direction; n = 1), neither HPV nor HIV were assessed (n = 37) nor a correlation was made between HIV and HPV (n = 17), specimen obtained for HPV testing was not anorectal (n = 1). The remaining 11 articles met the inclusion criteria and were selected for content analysis (Figure 1). Three of the 11 studies included in this review had a cohort study design and the remaining 8 were cross-sectional studies. Furthermore, our selected articles provided both cross-sectional [4,8,10,11,13,15,16,17] and longitudinal data [3,9,18] comparing HPV and HIV. Four of the 11 studies were conducted in the US and the other 7 in Buenos Aires (n = 1), Lima Peru (n = 1), Vancouver, Canada (n = 1), China (n = 2), Germany (n = 1) and Netherlands (n = 1) (Table 1). MSM were defined as individuals who were gay, bisexual, reported same sex encounters, and engaged in either insertive or receptive anal sex. The prevalence of HIV infection in MSM across all 11 studies ranged from 1.17% [1], to 85% [5]. HIV information was collected by self-report, ELISA with Western Blot confirmation, non-specified test on dried blood spot specimens, immunofluorescence and PCR confirmation, and non-specified HIV antibody detection test. Anal HPV prevalence among MSM across all 11 studies ranged between 13.7% [14] and 98% [5]. High risk oncogenic genotypes were assessed in all 11 research studies and testing methods for HPV included PCR + restriction fragment length polymorphism, line blot assay, Tellgenplex HPV DNA test, Linear Array line blot, linear array probe, suspension bead array method, hybrid capture II, cytology, dot blot hybridization, and SPF LIPA HPV PCR. Specimens for HPV testing were derived from perianal swabs, anal swabs, punch biopsies, digital rectal examination, anorectal swabs, and rectal swabs. With respect to the role of HPV in HIV acquisition, only one [1] of the 11 selected studies assessed this relationship in HIV-uninfected MSM. Among MSM 57% were infected with HPV, 26% with high risk HPV and 26% with low risk HPV. Of 1409 participants who contributed 4375 person years of follow-up, 51 sero-converted, leading to an HIV acquisition rate of 1.17 per 100-person years. The authors reported an unadjusted hazard ratio (uHR) of 2.8 (95% Confidence Interval (CI) 1.04-7.4, *p* = 0.04) in the association between having one type of HPV and HIV sero-conversion. Similarly, an uHR of 3.6 (95% CI 1.5-8.4, *p* = 0.004) and adjusted hazard ratio (aHR) of 3.5 (95% CI 1.2-10.6, *p* = 0.02) were reported in the association between having 2 or more types of HPV and HIV sero-conversion. Other variables that showed a statistically significant relationship with HIV sero-conversion were unprotected receptive anal intercourse with HIV-unknown partners (adjusted HR of 7.1, 95% CI 2.8-18.2, *p* < 0.0001) and methamphetamine use (adjusted HR of 4.6, 95% CI 1.7-12.0, *p* = 0.002). The same authors also found some evidence that abnormal anal cytology (Atypical Squamous Cells of Unidentified Significance (ASCUS)) was associated with HIV sero-conversion (HR 2.9, 95% CI 1.1-7.5, *p* = 0.03).

**Table 1:** Select characteristics of studies identified in the literature review, 1998–2012.

Author	Type of study	Country	(N)	HIV Prevalence	HPV Prevalence	HPV types tested
Pando *et al.*	x sectional	Argentina	496	17.30%	83.5%	36
Quinn, *et al.*	x sectional	Peru	105	36.50%	77.1%	7
Zhang *et al.*	x sectional	China	302	9.90%	13.7%	26
Gilbert *et al.*	x sectional	Canada	268	24.10%	62.3%	4
Gao *et al.*	x sectional	China	602	8.50%	62.1%	26
Goldstone *et al.*	cohort	US	412	31%	Over 2 yrs: stayed HPV+ (38%); became HPV+ (13%);	13
Chin-Hong *et al.*	cohort	US	1409	HIV acquisition 1.17/100 person-yrs	56.8%	22
Kreuter, *et al.*	x sectional	Germany	53	85%	98%	37
Goldstone *et al.*	x sectional	US	597	27%	30% in HIV+ group, 14% in HIV- group	18
Goldstone, *et al.*	x sectional	US	124	27.00%	48%	19
VanDer Snoek, *et al.*	cohort	Nether-lands	258	6.60%	Group A 3rd visit: 34.9% 6th visit, 36.6%. Group B 3rd visit 43.2% 6th visit, 49.1%	26

### 2.2 Discussion

We reviewed published research studies about the role of HPV in HIV-acquisition among MSM. Overall and across all relevant studies, the prevalence of HPV infection was significantly higher in MSM compared to demographically similar non-MSM. Moreover, the prevalence of HIV infection was higher in MSM compared to non-MSM and the prevalence of HPV infection was higher in HIV-infected MSM compared to HIV-uninfected MSM. These findings are consistent with a significant body of literature underscoring the vulnerability of MSM compared to the general population to disproportionately higher rates of STIs including HIV and HPV. Although the relationship between most common STIs and HIV infection is well established in the literature, few studies directly address the relationship between HPV infection and HIV incidence. In the published studies that addressed HIV incidence and HPV infection, there are limitations to the interpretation and generalizability of the findings due to potential confounders. One [1] of the 11 selected articles for review examined the direct association between HPV and HIV-acquisition in MSM. The study reported a 3.62% HIV incidence rate of 1.17 per 100-person years. The study also found that HPV was independently associated with HIV sero-conversion. Additionally, infection with one or more genotypes of HPV significantly increased the risk of acquiring HIV infection. The biological plausibility of increased HIV acquisition due to HPV infection as discussed by the authors suggest that HPV-related lesions facilitate the aggregation of HIV-susceptible cells (T-cells and macrophages) at the site of the lesion making them more accessible to HIV. The authors also noted that HPV lesions cause disruptions in the normal anorectal anatomical barrier [1]. 

## 3. Conclusions

We identified several manuscripts which provide data highlighting an association between HPV and HIV infection among MSM. However, only one study examined the direct relationship between HPV infection and HIV acquisition over time in MSM. Additional studies are needed to further examine this relationship, and one such study is being undertaken in Peru at this time. Additional clinical studies of HPV vaccines and other STI interventions would be worthwhile to assess the impact of the reduction of HPV infection on HIV incidence.
